# Characteristics of the Northern Hemisphere cold regions changes from 1901 to 2019

**DOI:** 10.1038/s41598-023-30263-1

**Published:** 2023-03-08

**Authors:** Yutao Huang, Lijuan Zhang, Yongsheng Li, Chong Ren, Tao Pan, Wenshuai Zhang, Fan Zhang, Chunyang Li, Jiakai Gu, Jie Liu

**Affiliations:** 1 Heilongjiang Province Key Laboratory of Geographical Environment Monitoring and Spatial Information Service in Cold Regions, Harbin Normal University, Harbin, 150025 China; 2 Heilongjiang Climate Center, Harbin, 150030 China; 3 Key Laboratory of Land Surface Pattern and Simulation, Institute of Geographic Sciences and Natural Resources Research, CAS, Beijing, 100101 China

**Keywords:** Climate change, Cryospheric science

## Abstract

The accurate delineation of the spatial extent of cold regions provides the basis for the study of global environmental change. However, attention has been lacking on the temperature-sensitive spatial changes in the cold regions of the Earth in the context of climate warming. In this study, the mean temperature in the coldest month lower than − 3 °C, no more than 5 months over 10 °C, and an annual mean temperature no higher than 5 °C were selected to define cold regions. Based on the Climate Research Unit land surface air temperature (CRUTEM) of monthly mean surface climate elements, the spatiotemporal distribution and variation characteristics of the Northern Hemisphere (NH) continental cold regions from 1901 to 2019 are analyzed in this study, by adopting time trend and correlation analyses. The results show: (1) In the past 119 years, the cold regions of the NH covered on average about 4.074 × 10^7^ km^2^, accounting for 37.82% of the total land area of the NH. The cold regions can be divided into the Mid-to-High latitude cold regions and the Qinghai-Tibetan Plateau cold regions, with spatial extents of 3.755 × 10^7^ km^2^ and 3.127 × 10^6^ km^2^, respectively. The Mid-to-High latitude cold regions in the NH are mainly distributed in northern North America, most of Iceland, the Alps, northern Eurasia, and the Great Caucasus with a mean southern boundary of 49.48° N. Except for the southwest, the entire region of the Qinghai-Tibetan Plateau, northern Pakistan, and most of Kyrgyzstan are cold regions. (2) In the past 119 years, the rates of change in the spatial extent of the cold regions in the NH, the Mid-to-High latitude, and the Qinghai-Tibetan Plateau were − 0.030 × 10^7^ km^2^/10 a, − 0.028 × 10^7^ km^2^/10 a, and − 0.013 × 10^6^ km^2^/10 a, respectively, showing an extremely significant decreasing trend. In the past 119 years, the mean southern boundary of the Mid-to-High latitude cold regions has been retreating northward at all longitudes. For instance, the mean southern boundary of the Eurasian cold regions moved 1.82° to the north and that of North America moved 0.98° to the north. The main contribution of the study lies in the accurate definition of the cold regions and documentation of the spatial variation of the cold regions in the NH, revealing the response trends of the cold regions to climate warming, and deepening the study of global change from a new perspective.

## Introduction

Cold regions can be broadly defined as regions where the natural environment and human activities are significantly affected by low temperatures^1^. Cold regions, as essential parts of the Earth’s ecosystem^2^, are mainly distributed at high latitudes or high elevations on Earth^3^. The change in cold regions has major effects on water resources and landscape scale ecology^4^; these, in turn, have important effects on variations in the global environment^5–8^. Numerous environmental factors in cold regions that are extremely sensitive to climate change, such as permafrost, snow, and glaciers have been emerging popular research areas in recent years^9,10^. Studies have shown that permafrost area^11,12^, snow cover ^13^, and sea ice areas ^14^ in the NH have decreased significantly. Since the last century, the mean annual surface temperature of the Earth has increased by 1.09 °C^15^. However, the most severe warming has occurred in the Mid-to-High latitude of NH ^16–19^. The Arctic has warmed at twice the global average^20^. Nevertheless, the response of cold regions to climate warming on a global scale has not received adequate attention. There is a lack of relevant studies that reveal the change in the spatial extent of the cold regions in the NH on the scale of long-term climate change. Clearly defining the spatial extent and changing the rules of cold regions is an important basis for maintaining global ecological security and formulating national development plans.

Köppen^21^ first defined indicators of a cold region as an area having the mean temperature in the coldest month no greater than − 3 °C and no more than 4 months with a monthly mean temperature higher than 10 °C. Bates and Bilello^22^ put forward classification criteria for cold regions based on the mean temperature of the coldest month lower than 0 °C, the maximum snow depth observed on the ground of more than 0.3 m, the mean freezing period of rivers and lakes of more than 100 days per year, and freezing depth of at least 1 year in every 10 years greater than 0.3 m. Gerdel^23^ pointed out that the southern boundary of a cold region can be determined by having a soil freezing depth of 0.15 m or 0.3 m.

Yang^24^ proposed the classification criteria for cold regions in China, including the temperature of the coldest month lower than − 3 °C, no more than 4 months with a monthly mean temperature higher than 10 °C, the freezing period for rivers and lakes of more than 100 days, and receiving more than 50% of all precipitation in a frozen form. Yang et al.^25^ also appended the cumulative temperature between 500 and 1000 °C and annual mean snow days of 30 days based on the above indicators to calculate the cold regions of China. Chen et al.^26^ simplified the division method proposed by Yang^24^ and put forward three indicators of cold regions, including having a mean temperature in the coldest month lower than − 3 °C, no more than 5 months having a mean temperature over 10 °C, an annual mean temperature no higher than 5 °C. By contrast, Woo^27^ indicated that more than just temperature should be considered when defining a cold region; however, the water content in the form of snow or ice above or below the ground was also considered an indicator of a cold region. Studies on cold regions worldwide have mainly focused on defining the indicators of cold regions, and most of these studies are relatively old, while the research results on the spatial extent division of cold regions are very limited.

In summary, the definitions of indicators of cold regions have experienced constant updating and change, which evolved from the standard definition of a cold region based on temperature into definitions that consider the freezing process of water bodies and variations in the form of precipitation. Because data related to certain indicators is difficult to obtain on a global scale, the mapping of subtle divisions is currently lacking for the distribution of cold regions in the NH. The cold regions of the NH change with ongoing global warming. Nevertheless, little relevant research on the topic has been published. Hence, using the centennial CRUTEM data^28^ and three indicators to define a cold region: having a mean temperature in the coldest month lower than − 3 °C, no more than 5 months with temperatures averaging over 10 °C, and an annual mean temperature no higher than 5 °C. The study quantitatively analyzed the distribution and variation trends of cold regions in the NH during the past 119 years. In addition, the study provides a basis for the study of global change, especially global environmental change.

## Results

### Variation characteristics of the NH cold regions

#### Area of the NH cold regions

**Figure 1:**
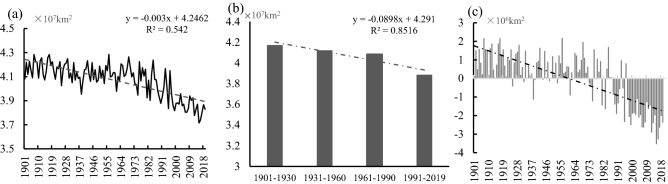
(**a**) Interannual and (**b**) tri-decadal variations and (**c**) anomalies in the spatial extent of the cold regions in the NH from 1901 to 2019.

The analysis results of the spatial extent of cold regions in the NH from 1901 to 2019 are shown in Table 1 and Fig. 1a. In 119 years, the mean spatial extent of the NH cold regions was about 4.074 × 10^7^ km^2^, accounting for 37.82% of the total land area in the NH. The rate of changes in the spatial extent of the cold regions in the NH was − 0.030 × 10^7^ km^2^/10 a from 1901 to 2019, showing an extremely significant decreasing trend (*P* < 0.001). The spatial extent of the cold regions declined continuously with time when comparing the four climatic periods defined as Period 1 (1901–1930), Period 2 (1931–1960), Period 3 (1961–1990), and Period 4 (1991–2019) continued to decline over time. The spatial extent of this region has declined significantly in the last 30 years (Period 4) and decreased by 6.88% compared with that in Period 1, showing a significant shrinkage (Fig. 1b).

**Table 1:** The mean area of the cold regions and its variation in the NH from 1901 to 2019.

Years	Area (10^7^ km^2^)	The land area of the NH (%)	Trend (10^7^ km^2^/10a)
1901–2019	4.074	37.820	− 0.030**
1901–1930	4.176	38.767	− 0.002
1931–1960	4.128	38.322	0.015
1961–1990	4.092	37.987	− 0.046
1991–2019	3.886	36.075	− 0.077**

“*” and “**”: P < 0.01 and 0.001, respectively.

The spatial extent of the NH cold regions exhibited a turning point from positive to negative anomalies relative to its 1901–2019 mean in 1985 (Fig. 1c). From 1901 to 1985, the mean area of this region was 4.135 × 10^7^ km^2^ and the rate of loss was − 0.011 × 10^7^ km^2^/10 a, showing a statistically significant declining trend (*P* < 0.01). From 1986 to 2019, the mean area of the cold regions in the NH was 3.901 × 10^7^ km^2^, with a rate of loss of − 0.072 × 10^7^ km^2^/10 a, showing an extremely significant declining trend (*P* < 0.001). Therefore, the rate of reduction of the spatial extent of this region has accelerated, which was 6.5 times that before 1985 (Table 1).

#### Spatial distribution of the NH cold regions

**Figure 2:**
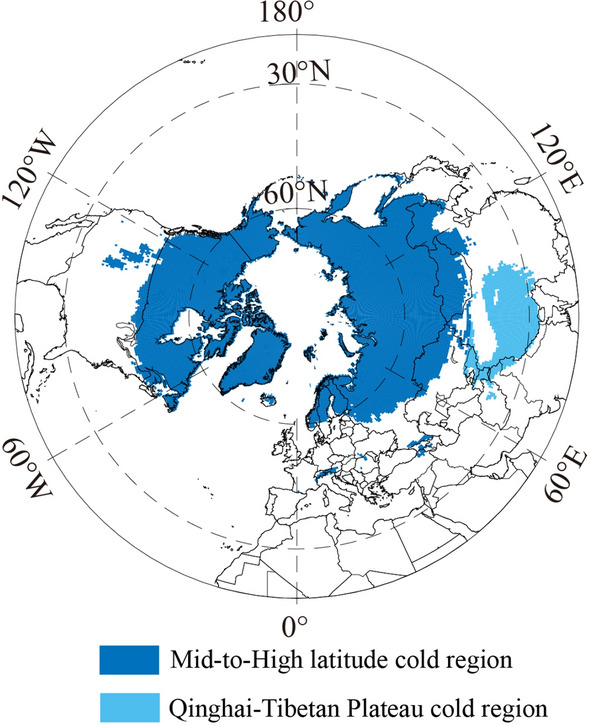
Spatial distribution of the cold regions in the NH from 1901 to 2019.

The spatial distribution of the cold regions in the NH from 1901 to 2019 is shown in Fig. 2. This region was mainly distributed in two areas, the Mid-to-High latitude cold regions and Qinghai-Tibetan Plateau cold regions. To clearly describe the spatial distribution and change of the cold regions in the NH, the Mid-to-High latitude cold regions and Qinghai-Tibetan Plateau cold regions were analyzed separately.

### Variation characteristics of the Mid-to-High latitude cold regions

#### Area of the Mid-to-High latitude cold regions

**Figure 3:**
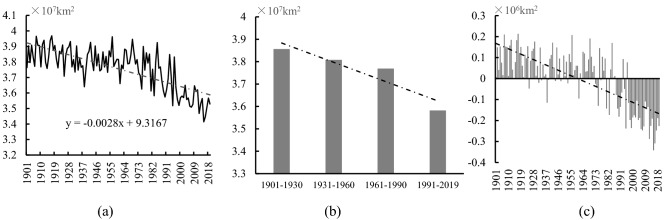
(**a**) Interannual and (**b**) tri-decadal variations and (**c**) anomalies in the spatial extent of the Mid-to-High latitude cold regions of the NH from 1901 to 2019.

In the past 119 years, the mean spatial extent of the Mid-to-High latitude cold regions was about 3.755 × 10^7^ km^2^, taking up 34.86% of the total land area in the NH. The rate of change in the spatial extent of this region was − 0.028 × 10^7^ km^2^/10 a (Fig. 3a), showing a very significant declining trend (*P* < 0.001). The spatial extent of this cold region decreased continuously between the four tri-decadal climatic periods 1–4 (Fig. 3b). When comparing Period 1 and Period 4, the cold regions have decreased by 0.275 × 10^7^ km^2^, which is 7.13% of the cold regions in Period 1 (Table 2).

**Table 2:** Variations of the spatial extent and the southern boundary of the Mid-to-High latitude cold regions from 1901 to 2019.

Years	Cold regions			Southern boundary	
	Area (10^7^ km^2^)	The land area of the NH (%)	Trend (10^7^ km^2^/10 a)	The mean latitude (°N)	Trend (°/10 a)
1901–2019	3.755	34.862	− 0.028**	49.48	0.144**
1901–1930	3.857	35.801	− 0.001	48.95	0.010
1931–1960	3.808	35.351	0.014	49.23	-0.082
1961–1990	3.769	34.988	− 0.042**	49.42	0.230
1991–2019	3.582	33.254	− 0.070**	50.35	0.306

“*” and “**”: P < 0.01 and 0.001, respectively.

The anomalies in the spatial extent of the Mid-to-High latitude cold regions from 1901 to 2019 are shown in Fig. 3c. In 1985, a turning point from a positive to a negative anomaly was observed. The spatial extents of the Mid-to-High latitude cold regions were 3.819 × 10^7^ km^2^ and 3.596 × 10^7^ km^2^ in the periods of 1901–1985 and 1986–2019, respectively. After 1985, the spatial extent of this region decreased by 0.223 × 10^7^ km^2^. Meanwhile, the linear rates of shrinkage of the Mid-to-High latitude cold regions in 1901–1985 and 1986–2019 were calculated as − 0.011 × 10^7^ km^2^/10 a (*P* < 0.01) and − 0.066 × 10^7^ km^2^/10 a (*P* < 0.001), respectively, showing a significant declining trend. After 1985, this rate of loss accelerated significantly, being six times faster than before 1985.

#### Spatial distribution of the Mid-to-High latitude cold regions

The spatial distribution of the Mid-to-High latitude cold regions of the NH from 1901 to 2019 is shown in Fig. 2. From 1901 to 2019, this region was mainly distributed in Canada, Greenland, most of Iceland, Alaska, and the Rocky Mountains in the United States, the Alps in central Europe, northern Eurasia, and the great Caucasus. In addition, the mean southern boundary of this region was around 49.48° N from 1901 to 2019 (Table 2), showing an irregular variation in longitude. Variation exists in the distribution of this region at different longitudes. The southern boundary of this cold region was the lowest at 106° W, at about 36.50° N. Furthermore, the southern boundary of the cold regions was around 60° N at 15.75° E, with a difference of nearly 23.5° in latitude. In general, the southern boundary of the cold regions was the furthest south in eastern Eurasia and the northern United States, and the furthest north in northwestern Eurasia.

**Figure 4:**
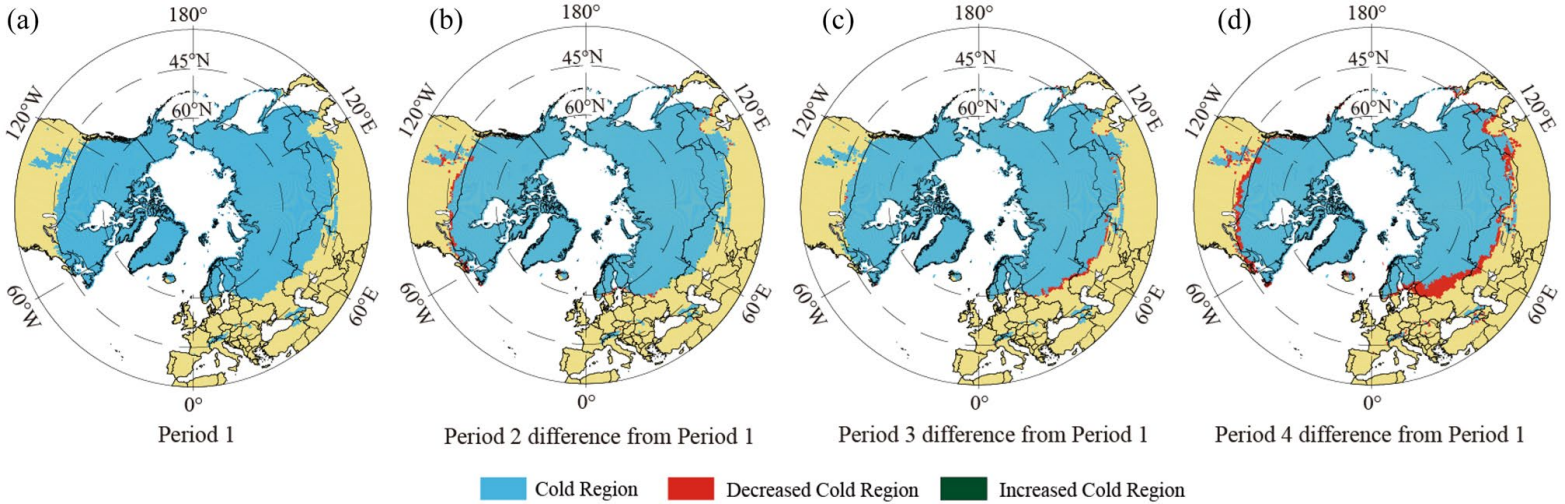
Spatial distribution and variation of the Mid-to-High latitude cold regions of the NH from 1901 to 2019.

Compared with Period 1, the spatial extent of the Mid-to-High latitude cold regions decreased by 0.049 × 10^7^ km^2^ in Period 2; the decrease mainly occurred along the border between Canada and the United States (at about 45° N), in the middle Rocky Mountains of the western United States, the south of Norway, Sweden, and Finland, eastern Estonia, western Russia, and Northeast China (Fig. 4a,b). In addition, there were sporadic increases in Eurasia, covering a total of 0.009 × 10^7^ km^2^. The mean southern boundary of the cold regions moved northward by around 0.28° globally (Fig. 4b). The spatial extent of this region in Period 3 was 0.088 × 10^7^ km^2^ smaller than in Period 1, with the decrease mainly distributed in southwest Russia and northern Kazakhstan (Fig. 4c). Yet the cold regions locally expanded in South Mongolia and Northeast China, the Rocky Mountains and northeastern America, south Sweden, and south Finland. The mean southern boundary of the cold regions moved northward by about 0.47°. Compared with Period 1, the Mid-to-High-latitude cold regions experienced a northward retreat of the southern boundary along almost all longitudes on a centennial scale in Period 4 (Fig. 4d). There is no need for the NH specification, as the whole study is about the NH. The southern boundary of this region in North America moved northward by 0.98° and that of Eurasia moved northward by 1.82°. In addition, the southern boundary of the cold regions in western Russia and northern Kazakhstan between 20° E and 62° E moved northward by 1.51°.

### Variation characteristics of the Qinghai-Tibetan Plateau cold regions

#### Area of the Qinghai-Tibetan Plateau cold regions

**Figure 5:**
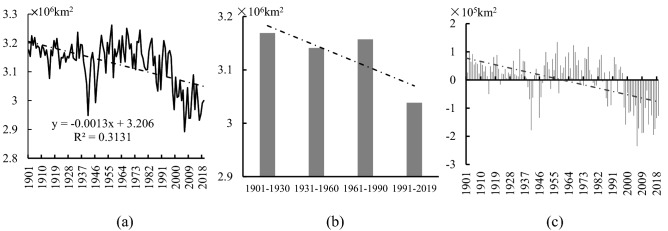
(**a**) Interannual and (**b**) tri-decadal variations and (**c**) anomalies in the spatial extent of the cold regions in the Qinghai-Tibetan Plateau from 1901 to 2019.

In the past 119 years, the mean spatial extent of the cold regions in the Qinghai-Tibetan Plateau was 3.127 × 10^6^ km^2^, accounting for about 2.09% of the total land area in the NH (Table 3, Fig. 5a). From 1901 to 2019, the change rate of the cold regions in the Qinghai-Tibetan Plateau was − 0.013 × 10^6^ km^2^/10 a, showing an extremely significant declining trend (*P* < 0.001). The spatial extent of the cold regions in the Qinghai-Tibetan Plateau in the four climatic periods showed a declining trend. Compared with Period 1, the spatial extent of the cold regions in the Qinghai-Tibetan Plateau decreased by 0.131 × 10^6^ km^2^ in Period 4, accounting for 4.13% of the cold regions in Period 1 (Fig. 5b).

**Table 3:** The mean area variation of the cold regions in the Qinghai-Tibetan Plateau from 1901 to 2019.

Years	Mean area (10^6^ km^2^)	The land area of the NH (%)	Trend (10^6^ km^2^/10 a)
1901–2019	3.127	2.903	− 0.013**
1901–1930	3.173	2.942	− 0.001
1931–1960	3.145	2.916	0.001
1961–1990	3.165	2.931	− 0.003**
1991–2019	3.053	2.821	− 0.007**

“*” and “**”: P < 0.01 and 0.001, respectively.

The spatial extent of the Qinqhai-Tibetan Plateau cold regions varied irregularly until the mid-1990s (1994) when there was a turning point from a positive anomaly to a negative anomaly (Fig. 5c). The spatial extent of the cold regions in the Qinghai-Tibetan Plateau in the periods of 1901–1994 and 1995–2019 were 3.156 × 10^6^ km^2^ and 3.020 × 10^6^ km^2^ respectively, with the rate of loss of − 0.003 × 10^6^ km^2^/10 a (*P* > 0.05) and − 0.054 × 10^6^ km^2^/10 a (*P* < 0.01), respectively. The spatial extent of the cold regions in the Qinghai-Tibetan Plateau after 1994 declined by 4.31% compared with that before. Moreover, the rate of decrease was 18 times faster than before 1994.

#### Spatial distribution of the Qinghai-Tibetan Plateau cold regions

**Figure 6:**
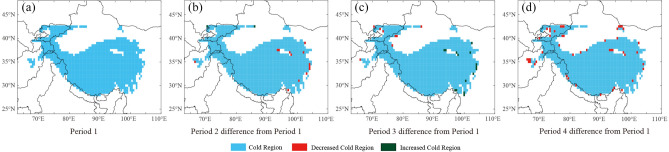
Spatial distribution and variation of the Qinghai-Tibetan Plateau cold regions from 1901 to 2019.

In terms of the average conditions, except for certain areas in the southeastern Qinghai-Tibetan Plateau (covering about 2.588 × 10^5^ km^2^) from 1901 to 2019, the cold regions of the Plateau almost covered the entire region of the Qinghai-Tibetan Plateau, including the western part of the Plateau in northern Pakistan and most of the region in Kyrgyzstan (about 7.846 × 10^5^ km^2^) (Fig. 2). The spatial extent of the cold regions declined by 0.028 × 10^6^ km^2^ in Period 2, compared with Period 1 (Fig. 6a,b); this decline was mainly distributed in the eastern Qinghai-Tibetan Plateau and a small part of the western Hindu Kush (Fig. 6b). The spatial extent of the cold regions increased by 0.008 × 10^6^ km^2^ from Period 1 to Period 3, showing a pattern of decreasing in the west and increasing in the east (Fig. 6c). Compared with Period 1, the cold regions that shrank in size in Period 4 were scattered around the Plateau and on the east and west sides of the Qaidam Basin in the northeastern part of the Plateau (Fig. 6d).

### Cold regions in Russia, China, and the United States

To further elaborate on the characteristics of variations in cold regions, the present study selected Russia, China, and the United States as representatives to analyze changes in cold regions in a specific country.

**Figure 7:**
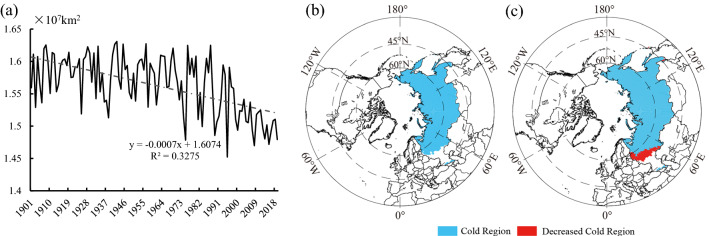
Area and spatial variations of the cold regions in Russia from 1901 to 2019. (**a**) The time change from 1901 to 2019; (**b**) Spatial distribution for Period 1; (**c**) the difference between Period 4 and Period 1.

Russia is the country with the largest reduced spatial extent of its cold regions in the NH. In the past 119 years (1901–2019), the spatial extent of this region in Russia was approximately 15.633 × 10^6^ km^2^, covering around 92.50% of the total land area in Russia (Table 4). The rate of change in the spatial extent of the cold regions in Russia from 1901 to 2019 was − 0.074 × 10^6^ km^2^/10 a, showing an extremely significant declining trend (*P* < 0.001) (Fig. 7a). From 1901 to 2019, most territories of Russia were located in the cold regions except southwest Russia (Fig. 7b,c). The cold regions of Russia declined by 0.701 × 10^6^ km^2^ in Period 4, compared with Period 1; this decline was mainly concentrated in the west of St. Petersburg in northwest Russia and Orenburg in southwest Russia. In addition, a small reduction was observed in Vladivostok in southeast Russia. Compared with Period 1, the southern boundary of the cold regions moved northward by 0.63° on average with a maximum northward movement of 2.96° in Period 4 (Fig. 7c).

**Table 4:** The mean area of the cold regions (10^6^ km^2^) and change rate (10^6^ km^2^/10 a) in Russia, the United States, and Northeast China from 1901 to 2019.

Years	Russia		The United States		Northeast China	
	Area	Trend	Area	Trend	Area	Trend
1901–2019	15.633	− 0.074**	2.341	− 0.045**	0.912	− 0.014**
1901–1930	15.874	0.055	2.565	− 0.012	0.963	0.003
1931–1960	15.803	0.042	2.335	0.047	0.941	0.001
1961–1990	15.666	− 0.126	2.354	− 0.072	0.913	− 0.029
1991–2019	15.173	− 0.227**	2.101	− 0.072	0.826	− 0.012

“*” and “**”: P < 0.01 and 0.001, respectively.

**Figure 8:**
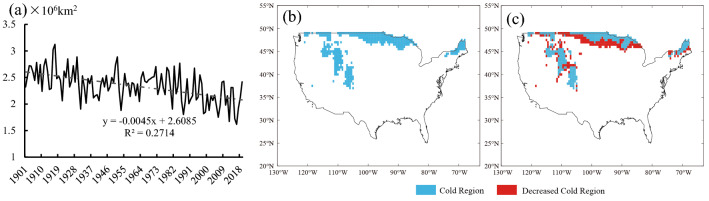
Area and spatial variations of the cold regions in the United States from 1901 to 2019. (**a**) The time change from 1901 to 2019; (**b**) Spatial distribution for Period 1; (**c**) the difference between Period 4 and Period 1.

The spatial extent of the cold regions in the United States, including Alaska, was about 2.341 × 10^6^ km^2^ in the past 119 years, accounting for 25.59% of the total land spatial extent of the United States (Table 4). From 1901 to 2019, the rate of change in the spatial extent of the cold regions in the United States was − 0.045 × 10^6^ km^2^/10 a, showing a very significant decreasing trend (*P* < 0.001) (Fig. 8a). From 1901 to 2019, the cold regions in the United States were mainly distributed in the northern parts of several states, Washington, Montana, South Dakota, Minnesota, Vermont, New Hampshire, and Maine as well as in most of North Dakota. In addition, there was a cold region in the Rocky Mountains (Fig. 8b,c). The spatial extent of the cold regions in the United States declined by 0.464 × 10^6^ km^2^, in Period 4 compared with Period 1, and the decrease was mainly distributed in northern Montana, northern South Dakota, southern Minnesota, and eastern Wisconsin. In addition, there were sporadic decreases in the Rocky Mountains. The mean southern boundary moved northward by 0.98° with a maximum northward movement of 1.50° (Fig. 8c).

**Figure 9:**
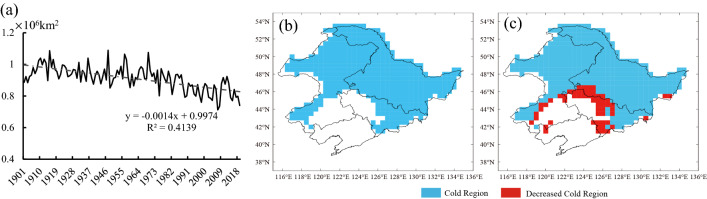
Area and spatial variations of the cold regions in Northeast China from 1901 to 2019. (**a**) The time change from 1901 to 2019; (**b**) Spatial distribution for Period 1; (**c**) the difference between Period 4 and Period 1.

The study chose Northeast China for analysis since China includes Northeast China and the Qinghai-Tibetan Plateau. In the past 119 years, the spatial extent of the cold regions in Northeast China was about 0.912 × 10^6^ km^2^, accounting for 9.50% of the total land area of China (Table 4). The rate of change in the spatial extent of the cold regions in Northeast China was − 0.014 × 10^6^ km^2^/10 a, showing an extremely significant decline (*P* < 0.001) (Fig. 9a). From 1901 to 2019, cold regions in Northeast China mainly included all of Heilongjiang, eastern Jilin, and eastern Inner Mongolia (Fig. 9b,c). Compared with Period 1, the spatial extent of the cold regions in Northeast China declined by 0.137 × 10^6^ km^2^ in Period 4 and the decrease was mainly distributed in southern Heilongjiang as well as the northern and southeast of Jilin. Moreover, the mean southern boundary of the cold regions moved 1.03° to the north with a maximum movement of 1.90° to the north (Fig. 9c).

## Discussion

At present, the cold regions in the NH are most commonly defined and are mainly divided based on the standards proposed by Bates and Bilello^22^, Woo^27^, and Parker^29^. Bates and Bilello^22^ considered that all the continents north of 40° in the hemisphere are located in cold regions and take up nearly half of the land area. Woo^27^ believed that this region covers almost all the land north of 40° N, including mountains and plateaus. In addition, Parker^29^ held the opinion that this region includes the Arctic, the Subarctic, and some areas further south. The results of the present study show that the mean area of the cold regions in the NH covers about 4.074 × 10^7^ km^2^ accounting for 37.82% of the total land area in the hemisphere. From 1901 to 2019, the cold regions in the hemisphere were mainly distributed in the Mid-to-High latitude cold regions and the Qinghai-Tibetan Plateau cold regions, specifically in Canada, Greenland, most of Iceland, Alaska, and the Rocky Mountains in the United States, the Alps in central Europe, northern Eurasia, and the greater Caucasus. The mean southern boundary of the Mid-to-High latitude cold regions was mainly distributed around 49.48° N, showing an irregular variation with longitude. Moreover, the cold regions to the south of this latitude are mainly distributed in high-elevation areas. Compared with existing research, the results of the present study are more specific and accurate, demonstrating a clear southern boundary of the main cold regions.

The present study reveals the variations in the spatial extent and location of cold regions in the NH, the Mid-to-High latitude, and the Qinghai-Tibetan Plateau from 1901 to 2019. Also, the spatial extent and spatial variation of the cold regions in the four climatic periods 1901–1930, 1931–1960, 1961–1990, and 1991–2019 are compared in the paper. Compared with the existing research, the present paper more comprehensively and concretely reveals the variation characteristics of the cold regions in the hemisphere.

**Figure 10:**
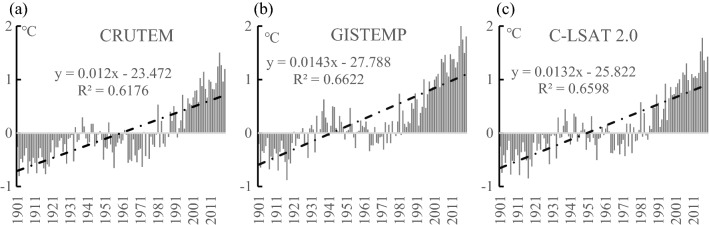
Variations of annual mean temperature in the NH from 1901 to 2019. (**a**) CRUTEM; (**b**) GISTEMP; (**c**) C-LAST2.0.

Although the three indicators related to temperature (mean temperature in the coldest month lower than − 3 °C, no more than 5 months over 10 °C, and an annual mean temperature no higher than 5 °C) were used in the study, the relationship between cold regions and temperature change has not yet been explicitly shown. To this end, CRUTEM^30^, GISTEMP^31^, and C-LAST2.0 data^32^ were used to analyze the variation of land temperature anomalies in the NH from 1901 to 2019 (Fig. 10a–c). In the past 119 years, the NH annual mean temperature showed a non-monotonous increase, with a shift from negative to positive anomalies around 1985. The results indicate that both the total area of the cold regions in the NH and the spatial extent of the Mid-to-High latitude cold regions changed in 1985, entering a period of rapid reduction of relative area. The total areas of cold regions in the NH and the Mid-to-High latitude from 1901 to 2019 are consistent with the turning point of the annual mean temperature in the NH from 1901 to 2019. Moreover, the correlation coefficient of the annual mean temperature of the three data sets in the NH with the spatial extent of the cold regions in the NH and the Mid-to-High latitude was all above 0.88, displaying an extremely significant negative correlation (*P* < 0.001) and suggesting that the annual mean temperature is an important indicator of the variation occurring in cold areas (Table 5).

**Table 5:** The correlation coefficient of the annual mean temperature in the NH with the cold extent.

	CRU	GISTEMP	C-LSAT
Cold regions in the NH	− 0.913**	− 0.891**	− 0.923**
Mid-to-High latitude cold regions	− 0.903**	− 0.881**	− 0.919**
Qinghai-Tibetan Plateau cold regions	− 0.731**	− 0.720**	− 0.705**

“*” and “**”: P < 0.01 and 0.001, respectively.

**Figure 11:**
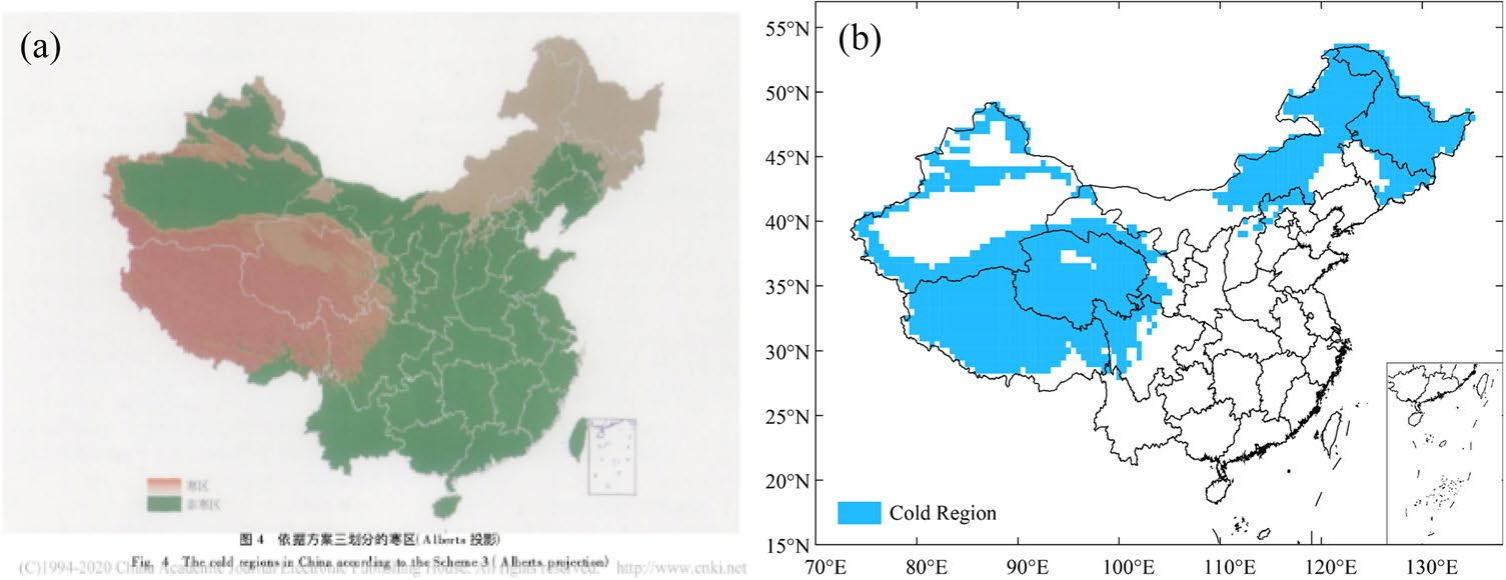
Comparison of spatial analysis results of the cold regions in China. (**a**) Research results of Chen (2005); (**b**) Analysis results of this study.

Yang et al.^25^ and Chen et al.^26^ analyzed cold regions in China from 1961 to 1989. Both authors considered that this region covers an area of 4.17 × 10^6^ km^2^ accounting for about 43% of the land area in China, including the Greater and Lesser Khingan Mountains in Northeast China, Changbai Mountains, Sanjiang Plain, Hexi Corridor, most mountainous areas in Xinjiang, and Qinghai-Tibetan Plateau (Fig. 11a). The spatial extent of the cold regions in China from 1961 to 1989 was 4.023 × 10^6^ km^2^ as defined in the present study, which is consistent with the results of Yang^25^ and Chen^26^, accounting for 41.9% of the land area in China (Fig. 11b). Compared with the existing research results, a slight difference exists in the northern Qinghai-Tibetan Plateau. The reason for the difference is that the data used was different. Chen used data from 571 surface meteorological stations in China while the present study used a data grid of 1696 cells. Therefore, the spatial resolution of the cold regions analyzed in the paper is higher than previous research results.

Compared with the existing studies, the paper further analyzes the spatial extent and spatial changes of cold regions in China in the past 119 years from 1901 to 2019, and in the four climatic periods described above. Meanwhile, Russia, the United States, and China are taken as examples to analyze the spatial extent, spatial distribution, and variation of cold regions in the three countries. The study does not specifically analyze the characteristics of variation in cold regions in other countries which are still in need of research.

The indicators of the cold regions division in China and abroad range from Koppen^21^, Bates and Bilello^22^, Gerdel^23^, Yang et al.^25^, Chen et al.^26^ to Woo^27^. The indicators gradually developed from simple temperature indicators to indicators reflecting the growth state of plants, the process of water freezing, and the change of precipitation form, considering that water exists in the form of snow or ice above or below ground. The study needs long-term series data support because the time scale is from 1901 to 2019. It is difficult to obtain indicators reflecting the growth state of plants, the process of water freezing, and the form of precipitation over that period and in general. In addition, scholars have proposed different definitions of cold regions. However, a comprehensive comparison shows that, in addition to the indicators related to temperature, other indicators such as those related to the process of water freezing and the form of precipitation are also in essence related to temperature. Hence, based on the availability and completeness of the data, the simplified indicators proposed by Chen et al.^26^ are used in the present paper to analyze the spatiotemporal variation characteristics of the cold regions in the NH in the centennial series.

Time series of local monthly average temperatures (gridded datasets) and global averages are produced by several organizations, with the product of the Hadley Centre and the Climatic Research Unit (CRU) being one of the most widely cited. However, there are uncertainties in the CRU data set^33^. First, observation coverage in developing countries and regions such as Asia is insufficient, and the data coverage rate was low before 1950. Second, insufficient attention has been paid to the impact of urbanization. Therefore, the results of the warming trend may be underestimated.

## Conclusions

1. The spatial extent of the cold regions in the NH from 1901 to 2019 is about 4.074 × 10^7^ km^2^, accounting for 37.82% of the total land area in the NH. In addition, the rate of change in the spatial extent of the cold regions was − 0.030 × 10^7^ km^2^/10 a (*P* < 0.001). In the past 119 years, the spatial extent of this region reached a turning point in 1985. The rate of loss after 1985 was 6.5 times that before 1985.
2. From 1901 to 2019, the mean spatial extent of the Mid-to-High latitude cold regions was about 3.755 × 10^7^ km^2^, accounting for 34.86% of the total land area in the hemisphere. In addition, the rate of the change in spatial extent was − 0.028 × 10^7^ km^2^/10 a (*P* < 0.001). In the past 30 years (1991–2019), compared with Period 1 (1901–1930), the spatial extent of this region fell by 7.13%. The mean southern boundary of the cold regions retreated northward at almost all longitudes.
3. The mean spatial extent of the cold regions in the Qinghai-Tibetan Plateau and its surrounding areas was 3.127 × 10^6^ km^2^ with a rate of change of − 0.013 × 10^6^ km^2^/10 a in the past 119 years (*P* < 0.001). The spatial extent of the cold regions declined in the past 119 years, scattered around the Qinghai-Tibetan Plateau and on the east and west sides of the Qaidam Basin, which lies northeast of the Qinghai-Tibetan Plateau.
4. Russia is the country with the largest reduction in the spatial extent of the cold regions in the NH. Over the past 119 years, the spatial extent of cold regions in Russia, the United States, and Northeast China has shown a significant declining trend. In addition, the mean southern boundary of the cold regions moved northward by 0.63°, 0.98°, and 1.03° in these regions, respectively.

## Materials are methods

### Datasets

This study deals with the centennial time scale. So far, there are three famous global centennial land near-surface air temperature datasets. They are the CRUTEM established by the Climatic Research Unit of the University of East Anglia^34^, the Global Historical Climatology Network Dataset (GHCN-V3) established by the National Climatic Data Center of the United States^35^, and the Goddard Institute for Space Studies Dataset (GISTEMP) established by the National Aeronautics and Space Administration^36^. Among them, GHCN-3 and GISTEMP datasets only provide century-scale monthly temperature anomaly data, not monthly average temperature data, and only CRUTEM data contain monthly average temperature data. In this paper, the cold regions division is mainly based on the monthly average temperature calculation. Hence, the temperature data used in this paper comes from the CRUTEM dataset. This provides a dataset with comprehensive, high-resolution, and continuous surface meteorological elements reconstructed by the Climatic Research Unit of the University of East Anglia, UK by integrating several databases. The dataset covers all global land, deserts, and plateaus without missing measurements. At the time that this research was conducted, the latest version of CRUTEM is the CRU-TS 4.06 dataset. The time range extends from January 1901 to December 2019 with a horizontal grid resolution of 0.5° × 0.5°. The dataset has been widely used to analyze variations of the global surface and upper air meteorological elements^37,38^. In the present paper, monthly grid temperature data of the dataset was selected on a time scale from 1901 to 2019. The data are registered and were downloaded from https://crudata.uea.ac.uk/cru/data. Moreover, 54,023 grid points represent the land area of the NH.

## Methods

### Cold regions definition method and calculation

Among the proposed definitions of cold regions, Chen et al.’s definition would be relatively rigorous^26^, since the boundary and distribution of the cold regions were accordant to those of permafrost, glaciers, snow cover, vegetation distribution, and climate regionalization. Thus, we selected the mean temperature in the coldest month lower than − 3 °C, no more than 5 months over 10 °C, and an annual mean temperature no higher than 5 °C to define the cold regions. Based on the monthly land temperature data of the NH provided by CRU, the annual area of the cold regions was calculated according to the cold regions definition method, and the mean values and interannual variation of the cold region in the NH, the Mid-to-High latitude, and the Qinghai-Tibetan Plateau from 1901 to 2019 were analyzed. Taking 30 years as one climate state, the mean values and interannual variation of cold regions in 1901–1930, 1931–1960, 1961–1990, and 1991–2019 were analyzed respectively. Then three countries, Russia, China, and the United States, are selected as representatives to analyze the changes in the cold regions in a specific country. In the analysis of the spatial distribution of the cold regions, based on the monthly average data combined with the cold regions definition method to judge the distribution of the cold region and show the spatial distribution of the mean value of the cold regions. When determining the average latitude for the southern boundary of the cold regions, the grid at each longitude is determined by the continuity of the cold regions as latitude increases. If the cold regions extend continuously to at least 8 grids in the south-north direction, the southernmost of these grids is the southern boundary of the cold regions.

#### Trend analysis

Trend analysis refers to the change analysis of a certain element showing a continuous increase or decreases over a long period. The linear least squares regression is applied to analyze cold regions change. A univariate linear regression equation of the cold regions variable (y) and the corresponding time (x) was established^39^:

1 $$y=ax+b i=\mathrm{1,2},\dots ,n,$$

where *a* is the linear regression coefficient indicating the rate of change in the spatial extent of the cold regions. The positive or negative value of* a* indicates that the spatial extent of the cold regions is increasing or decreasing over time.

### Pearson correlation coefficient

Pearson correlation coefficient method is a statistical method to accurately measure the correlation of the relationship between two variables If n pairs of observations of two-dimensional climate variables $(x_{1} ,y_{1} ),(x_{2} ,y_{2} )...,(x_{n} ,y_{n} )$ are set, the Pearson correlation coefficient r is^40^:

2 $$r=\frac{\sum_{i=1}^{n}\left({x}_{i}-\overline{x }\right)\left({y}_{i}-\overline{y }\right)}{\sqrt{\sum_{i=1}^{n}{\left({x}_{i}-\overline{x }\right)}^{2}}\sqrt{\sum_{i=1}^{n}{\left({y}_{i}-\overline{y }\right)}^{2}}},$$

where $\overline{x }$, $\overline{y }$ are the mean values of sequence *x* and *y*, respectively. The range of *r* is 0 ≤|*r*|≤ 1. *r* is positive for a positive correlation while *r* is negative for an inverse correlation.

### Determination of transition years

The moving average method is also called the rolling average method^41^. The transition years from positive to negative (or negative to positive) anomalies of cold area extent and temperature are defined as the years when the 5-year moving average of the anomaly changes sign from positive to negative (or negative to positive).

## Data Availability

Correspondence and requests for data should be addressed to L. J. Zhang (zhlj@hrbnu.edu.cn) or Y. T. Huang (huangyutao0128@hrbnu.edu.cn).

## References

[1] Gelfan AN, Motovilov YG (2009). Long-term hydrological forecasting in cold regions: Retrospect, current status and prospect. Geogr. Compass..

[2] Shen H (2015). Colde Regions Science and Marine Technology.

[3] Du JY, Watts JD, Jiang LM, Lu H, Chen X, Duguay C, Farina M, Qu YB, Kim Y, Kimball JS, Tarolli P (2019). Remote sensing of environmental changes in cold regions: Methods, achievements and challenges. Remote Sens..

[4] Wang, G. X., Zhang, Y. S. *Ecohydrology in Cold Regions: Theory and Practice*. (2016).

[5] Barandun M, Fiddes J, Scherler M, Mathys T, Saks T, Petrakov D, Hoelzle M (2020). The state and future of the cryosphere in Central Asia. Water Secur..

[6] Devoie É (2021). The Changing Influence of Permafrost on Peatlands Hydrology.

[7] Huang SB, Chang ZQ, Xie C, Tian BS (2020). Deformation monitoring of frozen soil in salt lake area based on SBAS-InSAR. Adv. Geosci..

[8] Jones DB, Harrison S, Anderson K, Shannon S, Betts RA (2021). Rock glaciers represent hidden water stores in the Himalaya. Sci. Total Environ..

[9] Aygün O, Kinnard C, Campeau S (2020). Impacts of climate change on the hydrology of northern midlatitude cold regions. Progr. Phys. Geogr. Earth Environ..

[10] Nie L, Lu J, Leiviskä T, Zhang L, Yu T (2021). Editorial: Assessment and adaptation to climate change impacts in cold regions. J. Water Clim. Change..

[11] Li T, Chen YZ, Han LJ, Cheng LH, Lv YH, Fu BJ, Feng XM, Wu X (2021). Shortened duration and reduced area of frozen soil in the NH. Innovation..

[12] Li G, Zhang M, Pei W, Melnikov A, Khristoforov I, Li R, Yu F (2022). Changes in permafrost extent and active layer thickness in the NH from 1969 to 2018. Sci. Total Environ..

[13] Mudryk L (2020). Historical NH snow cover trends and projected changes in the CMIP6 multi-model ensemble. Cryosphere.

[14] Thoman, R. L., Richter-Menge, J., Druckenmiller, M. L. Arctic report card 2020. Washington, D.C. (2020).

[15] IPCC. Summary for Policymakers. In Climate Change 2021: The Physical Science Basis. In *Contribution of Working Group I to the Sixth Assessment Report of the Intergovernmental Panel on Climate Change* (Cambridge University Press, 2021).

[16] DeBeer CM, Wheater HS, Carey SK, Chun KP (2016). Recent climatic, cryospheric, and hydrological changes over the interior of western Canada: A review and synthesis. Hydrol. Earth Syst. Sci..

[17] Easterling DR, Karl TR, Gallo KP, Robinson DA, Trenberth KE, Dai A (2000). Observed climate variability and change of relevance to the biosphere. J. Geophys. Res. Atmos..

[18] Kim SJ, Choi HS, Kim BM, Park SJ, Shim T, Kim JH (2013). Analysis of recent climate change over the Arctic using ERA-Interim reanalysis data. Adv. Polar Sci..

[19] Screen JA, Deser C, Simmonds I (2012). Local and remote controls on observed Arctic warming. Geophys. Res. Lett..

[20] Serreze MC, Barry RG (2011). Processes and impacts of Arctic amplification: A research synthesis. Glob. Planet Change..

[21] Köppen, W. *Das Geographische System der klimate*. (Gebrüder Bornträger, 1936).

[22] Bates, R. E. & Bilello, M. A. *Defining the Cold Regions of the Northern Hemisphere*. (1966)

[23] Gerdel, R. W. *Cold Regions Science and Engineering Monograph 1-A: Characteristics of the Cold Regions.* (1969).

[24] Yang, Z. *Research on Cold Regions Hydrology in China.* (Science Press, 1997) (**in Chinese**).

[25] Yang, Z., Liu, X., Zeng, Q. *Hydrology in Cold Regions of China*. (Science Press, 2000) (**in Chinese**).

[26] Chen RS, Kang ES, Ji XB, Yang JP, Yang Y (2006). Cold regions in China. Cold Reg. Sci. Technol..

[27] Woo M (2012). Permafrost Hydrology.

[28] Jones, P. D., Lister, D. H., Osborn, T. J., Harpham, C., Salmon, M., Morice, C. P. Hemispheric and large-scale land-surface air temperature variations: An extensive revision and an update to 2010. *J. Geophys. Res. Atmos.* **117** (2012).

[29] Parker DJ (2014). Floods.

[30] Harris I, Osborn TJ, Jones P, Lister D (2020). Version 4 of the CRU TS monthly high-resolution gridded multivariate climate dataset. Sci. Data..

[31] Lenssen N, Schmidt G, Hansen J, Menne M, Persin A, Ruedy R, Zyss D (2019). Improvements in the GISTEMP uncertainty model. J. Geophys. Res. Atmos..

[32] Sun W, Li Q, Huang B, Cheng J, Song Z, Li H, Dong W, Zhai P, Jones P (2021). The assessment of global surface temperature change from 1850s: The C-LSAT2.0 ensemble and the CMST-interim datasets. Adv. Atmos. Sci..

[33] Wen XY, Wang SW, Zhu JH, David V (2006). An overview of China climate change over the 20th century using UK UEA/CRU high-resolution grid data. Chin. J. Atmos. Sci..

[34] Morice CP, Kennedy JJ, Rayner NA, Jones PD (2012). Quantifying uncertainties in global and regional temperature change using an ensemble of observational estimates: The HadCRUT4 data set. J. Geophys. Res..

[35] Peterson TC, Vose RS (1997). An overview of the global historical climatology network temperature database. Bull. Am. Meteorol. Soc..

[36] Hansen J, Ruedy R, Sato M, Lo K (2010). Global surface temperature change. Rev. Geophys..

[37] Jones PD, Moberg A (2003). Hemispheric and large-scale surface air temperature variations: An extensive revision and an update to 2001. J. Clim..

[38] Wen X (2006). An overview of China climate change over the 20th century using UK UEA/CRU high resolution grid data. Chin. J. Atmos. Sci..

[39] Jia JP (2018). Statistics.

[40] Ji C, Zhang Y, Cheng Q, Li Y, Jiang T, Liang XS (2018). On the relationship between the early spring Indian’s sea surface temperature (SST) and the Tibetan Plateau atmospheric heat source in summer. Glob. Planet. Chang..

[41] Box GEP, Pierce DA (1970). Distribution of residual autocorrelations in autoregressive-integrated moving average time series models. J. Am. Stat. Assoc..

